# Resolving the structure and assembly of the honeybee silk heterotetrameric coiled coil

**DOI:** 10.1002/pro.70230

**Published:** 2025-07-23

**Authors:** Caitlin L. Johnston, Chacko Jobichen, Lyndall J. Briggs, Michelle Michie, Jian‐Wei Liu, Craig J. Morton, Andrew C. Warden, Tara D. Sutherland

**Affiliations:** ^1^ Health & Biosecurity Research Unit CSIRO Canberra Australian Capital Territory Australia; ^2^ Centre for Advanced Microscopy Australian National University Canberra Australian Capital Territory Australia; ^3^ School of Chemistry and Molecular Biosciences The University of Queensland St Lucia Queensland Australia; ^4^ Environment Research Unit CSIRO Canberra Australian Capital Territory Australia; ^5^ Australian Manufacturing and Materials Precinct CSIRO Parkville Victoria Australia

**Keywords:** coiled coil, coiled coil assembly, heterotetrameric, protein structure, silk

## Abstract

Coiled coil structures, first proposed by Crick in the 1950s, are protein structural motifs found across diverse biological systems. Honeybee silk was among the earliest identified coiled coils, with X‐ray diffraction studies in the 1960s revealing its characteristic helical packing. Decades of research have provided insights into silk composition and formation, yet the molecular details of its coiled coil assembly and final structure remained unresolved. In this study, we generated a structural model of the tetrameric coiled coil using AlphaFold and validated it with crosslinking mass spectrometry and medium‐resolution cryo‐electron microscopy. The model reveals that the four proteins (F1‐F4) adopt an antiparallel configuration in a defined clockwise arrangement (F1‐F3‐F2‐F4). Furthermore, we experimentally investigated the formation of this coiled coil complex using biochemical techniques, including blue‐native PAGE and circular dichroism spectroscopy. The sum of these experimental results and the structural predictions has allowed for the elucidation of key transitional steps in the assembly pathway, suggesting molecular interactions that may drive tetramer formation. These findings support a stepwise assembly model in which F2 and F4 form a stable core, F3 binds to the complex, and F1 initiates formation of the final, highly ordered structure. These structural insights establish a framework for understanding and directing coiled coil assembly, the fundamental building block of honeybee silk. By resolving this structure and its assembly process, this work lays the foundation for future rational design of functional sequences and materials with tailored properties.

## 1 INTRODUCTION

Silks are remarkable natural protein‐based materials produced and used by many insects for survival and protection. Among these insects, many bee, ant, and hornet species within the Aculeata group produce silk materials characterized by a coiled coil molecular structure, distinguishing them from the more commonly studied silks of spiders and silkworms (Sutherland et al. 2010). Coiled coil protein motifs are found throughout all kingdoms of life supporting a wide range of biological processes (Lupas et al. 2017; Truebestein and Leonard 2016). These protein structures are defined by two or more α‐helices that wind around each other to form a supercoil. Formation of the structure is driven by a seven‐residue repeat in the amino acid sequence (*abcdefg*) where the first (*a*) and fourth (*d*) residues generally are more apolar hydrophobic than the others and form the hydrophobic core of the coiled coil (Woolfson 2023). The fifth (*e*) and seventh (*g*) residues are often amino acids that are involved in electrostatic interactions between helices, and together with the hydrophobic interactions, define the specificity of the coiled coil oligomerization and overall structure.

An early study in the 1960s on honeybee (*Apis mellifera*) silk first revealed the characteristic coiled coil feature of these silk materials, with X‐ray diffraction patterns indicating a tetrameric structure (Atkins 1967; Rudall 1962). Subsequent studies have confirmed the structure in silks from other species of bees, ants, and hornets using techniques such as X‐ray diffraction, infrared spectroscopy, and solid‐state NMR (Kameda et al. 2005; Kameda et al. 2014; Kameda and Tamada 2009; Sutherland et al. 2007; Weisman et al. 2010). These coiled coil structures are widely regarded as core building blocks essential to the process of silk fiber formation. In honeybees, this process begins in the silk glands of the larvae, where the four silk proteins (AmelF1‐4) are synthesized and fold into coiled coils that assemble into ordered, birefringent structures named tactoids (Flower and Kenchington 1967). These structures reduce the flow viscosity of the silk protein solution, allowing the solution to pass through the spinneret and form the silk materials (Walker et al. 2015).

Coiled coils have clear and well‐defined structural constraints making them attractive for the de novo design of novel materials (Britton et al. 2024; Jorgensen and Chmielewski 2022; Woolfson 2023). Coiled coil silk gene sequences, encoding the four orthologous silk proteins from the honeybee, were first described in 2007 (Sezutsu et al. 2007; Sutherland et al. 2006; Sutherland et al. 2007). Subsequently, recognition that the size and sequence of the proteins were ideal for recombinant production and that the proteins self‐assembled into what appeared to be their native coiled coil structure in solution and retained this structure in the resulting materials drove an interest in their development for diverse applications, particularly in the biomedical and engineering fields. Silk proteins produced recombinantly in *E. coli* (Kambe et al. 2014; Maitip et al. 2015; Rapson et al. 2017; Weisman et al. 2010) have been fabricated into materials that are biocompatible and biodegradable and have good mechanical properties (Sutherland et al. 2018, 2019; Zhang et al. 2010). These materials have been used as passive scaffolds for reactive porphyrin compounds or silver nanoparticles to generate a range of functional materials such as oxygen and nitric oxide sensors; catalytic materials for fuel cells, remediation of toxicants, or for hydrogen production; and antimicrobial materials (Horgan et al. 2016; Musameh et al. 2018; Rapson et al. 2015; Rapson et al. 2017; Trueman et al. 2017). While their use as passive scaffolds highlights their versatility as structural materials, a detailed understanding of the coiled coil structure of these silks offers an opportunity for rational design of novel functionalities directly into these materials.

To fully harness the potential of these silks for rational design, an understanding of the atomic structure of the tetrameric coiled coil and its assembly process is essential. Current knowledge suggests the four silk proteins are modular in design, each containing a central alanine‐rich region of approximately 210 amino acids predicted to form the coiled coil structure, flanked by unstructured regions (Sezutsu et al. 2007; Sutherland et al. 2007). The alanine rich core has led to the suggestions that these structures may resemble tetrameric “Alacoils” with helices rotated to form a three‐residue *x‐da* type core, and axial stagger between helices (Sutherland et al. 2007). The high alanine content is consistent with data from early X‐ray scattering suggesting an unusually small superhelical radius of 5.2 Å (Atkins 1967; Rudall 1962). Based on the radius, the pitch in the bee silk was calculated at 140 Å. Later studies on hornet silk calculated a longer pitch of 172 Å (Kameda et al. 2014). Axial periods have been measured as 33.1 nm for bee silk and 34.4 nm for hornet silk (Fraser and Parry 2015; Kambe et al. 2014). Although these structural dimensions provide insights into the potential dimensions of the coiled coils, the exact sequence–structure relationship and the core packing interactions involved in the tetrameric silk complex remain unresolved.

In this study, we used AlphaFold to generate a predicted model of the tetrameric coiled coil structure of honeybee silk proteins and validated it experimentally with crosslinking mass spectrometry (XL‐MS) and a medium‐resolution cryo‐electron microscopy model. We also describe the assembly process, identifying key transitional steps that drive the formation of the coiled coil complex. These findings provide crucial insights into the structure and assembly of the tetrameric silk coiled coil, laying the groundwork for the rational design of new functionalities into these silk materials.

## 2 RESULTS

### 2.1 Validation of coiled coil tetramer silk model

**FIGURE 1:**
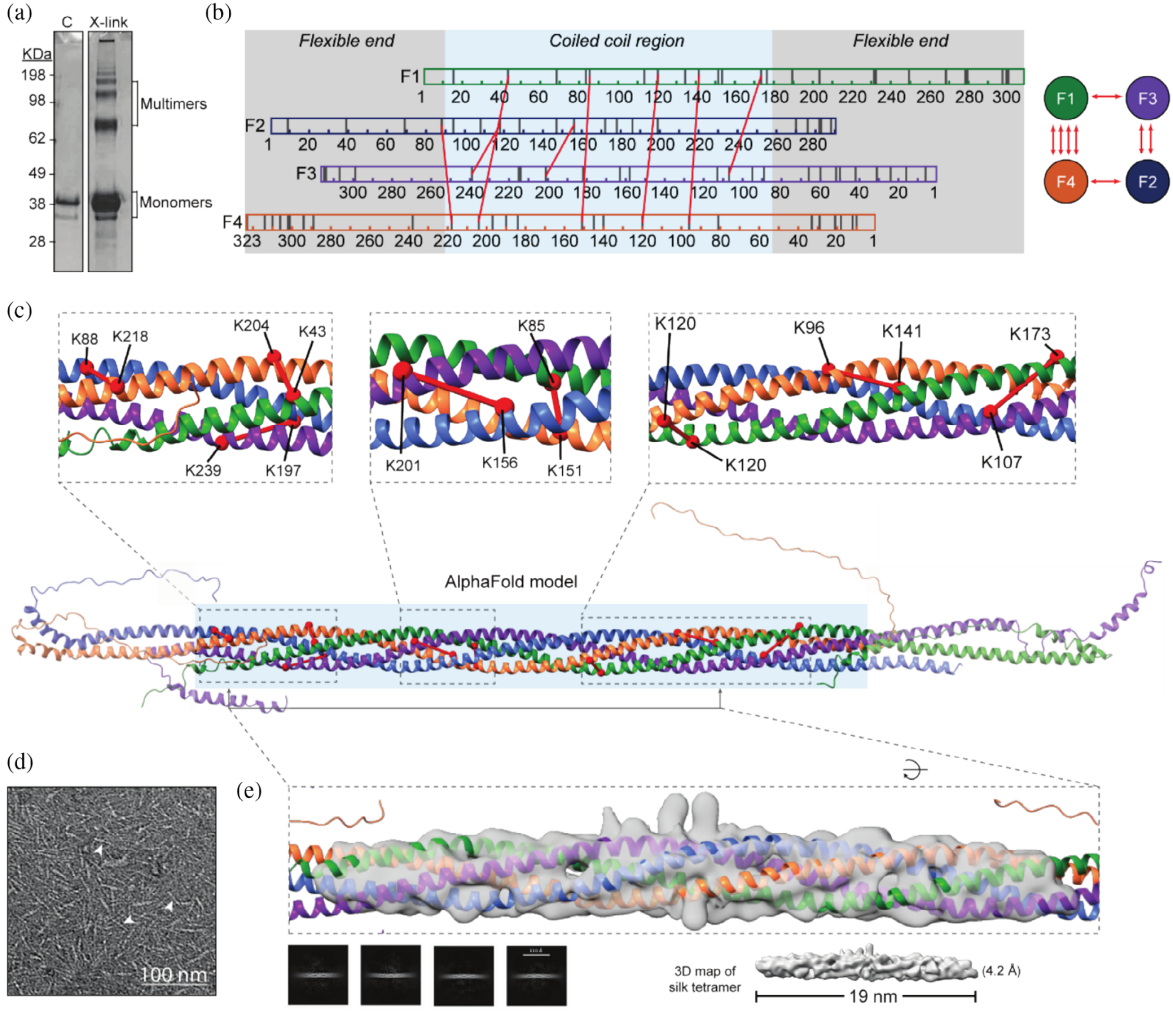
Validation of silk tetrameric coiled coil Alphafold model using crosslinking mass spectrometry and medium resolution cryo‐EM. (a) SDS‐PAGE gel of F1‐F4 protein mixtures before (c) and after (X‐link) crosslinking with BS^3^ crosslinker. (b) Linear and transverse maps of inter‐molecular BS^3^ crosslinks identified in the coiled coil regions of the F1‐F4 multimers. Gray lines indicate the positions of lysine residues within each silk protein. (c) Intermolecular BS^3^ crosslinks (<30 Å) mapped onto the AlphaFold2 predicted model within the coiled coil region. (d) Example transmission electron microscopy image of F1‐F4 particles (~25 nm). Arrows indicate examples of singular silk complexes. (e) Cryo‐EM analysis of silk tetramer; 2D Cryo‐EM classes, 3D map of silk tetramer alone and fit with the AlphaFold model.

AlphaFold2 predicted that the combined silk proteins (F1‐F4) form a heterotetrameric coiled coil structure (Figure 1). To validate this predicted model, we employed crosslinking mass spectrometry using the bifunctional cross‐linker BS^3^, which targets surface‐exposed primary amino groups, that is, lysine residues and the N‐termini. Each silk protein contains multiple lysines, with the predicted coiled coil region containing 44 lysine residues. The four silk proteins were combined under conditions known to promote coiled coil assembly, crosslinked, and separated via SDS‐PAGE (Figures 1a and S3, Supporting Information). Analysis of the crosslinked proteins by mass spectrometry identified both intra‐ and intermolecular crosslinks, with 70% of the lysine residues within the coiled coil region represented. A total of 28 unique intermolecular crosslinks were detected, of which 8 crosslinks were present multiple times within the predicted coiled coil region across the replicate experiments. These 8 crosslinks could be convincingly mapped onto the AlphaFold structure within the expected distance restraints of the linker (<30 Å) (Merkley et al. 2014), at predicted solvent‐exposed heptad positions (*b*, *c*, *e*, *f*, and *g*) (Figure 1b,c), and between predicted adjacent proteins. The other crosslinks were present in the poorly predicted ends and therefore could not be used to validate the model. These findings strongly support the predicted orientation, spatial arrangement, and alignment of the silk proteins within the AlphaFold predicted tetrameric coiled coil structure.

To further validate the model, we examined the silk complex using cryo‐EM. TEM screening of the F1‐F4 silk protein mixture revealed small rod‐shaped structures approximately 25 nm in length, consistent with the dimensions of the coiled coil domain of the model (24.3 nm) (Figures 1d and S4). These structures were subsequently visualized under a cryo‐EM microscope (Figure S5). Cryo‐EM data processing and subsequent 2D classification showed these structures are symmetric with continuous densities. The resulting 3D reconstruction revealed an elongated rod‐shaped complex with some variable α‐helical surface density visible across ~19 nm of the structure resolved at approximately 4.2 Å. The width of the map and the twisting of the chain densities were consistent with the predicted coiled coil geometry of the model, confirming the overall dimensions and arrangement of the tetramer. The variations in surface density likely reflect structural heterogeneity or flexibility within the coiled coil region. The terminal regions of the proteins could not be resolved in the density map. Together, the crosslinking data and cryo‐EM data support the predicted AlphaFold model, suggesting that the silk proteins form the described heterotetrameric coiled coil structure.

### 2.2 Structural analysis of the coiled coil region of the silk heterotetramer

**FIGURE 2:**
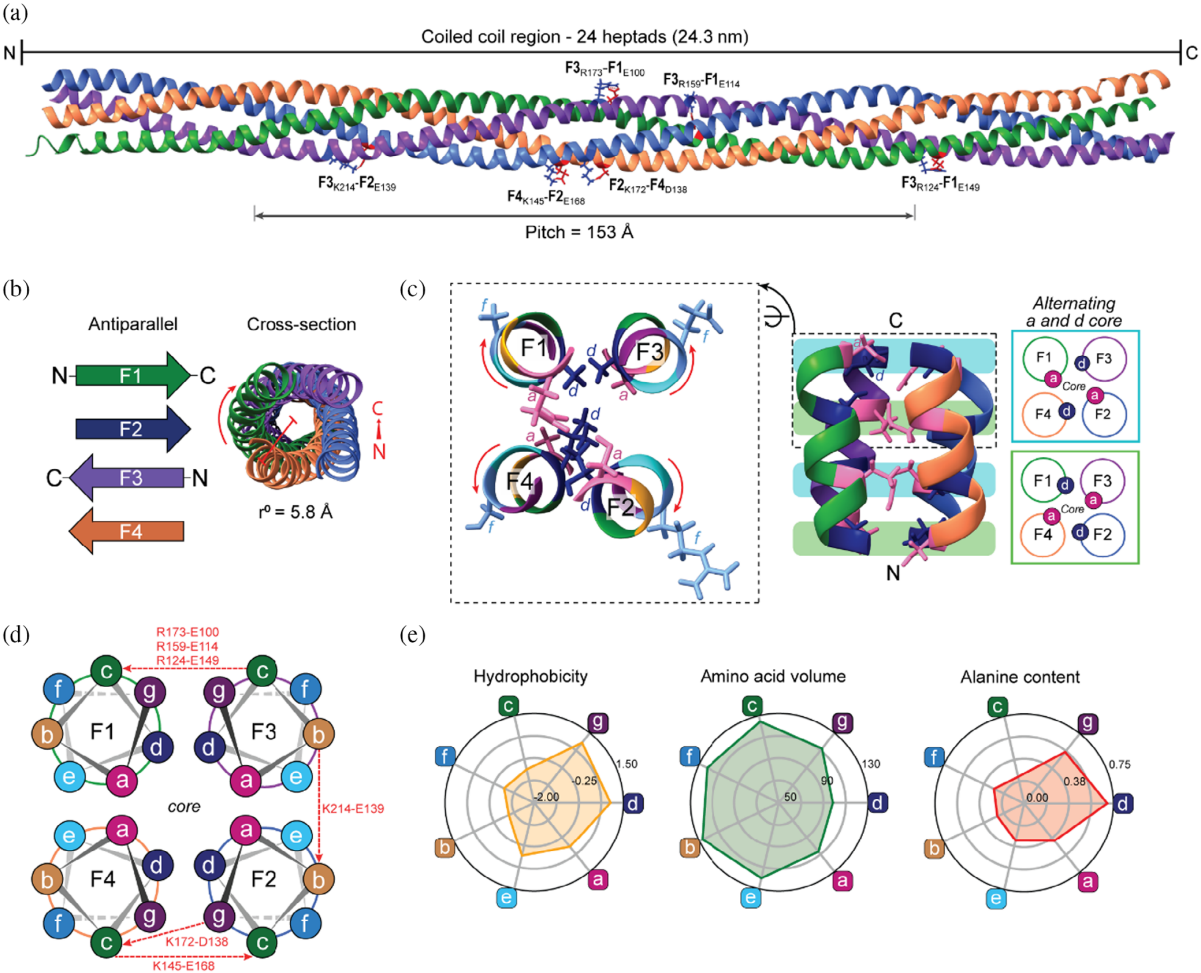
Analysis of coiled coil region of silk tetramer. (a) Coiled coil region of the silk tetramer showing all ionic interactions within this region. (b) Cross‐section and orientation of the silk proteins (F1‐F4) within the coiled coil structure. (c) Core packing of the coiled coil showing the alternating a and d core. (d) Schematic of cross‐section of the coiled coil structure showing position ionic interactions (e) Radar plots showing the average hydrophobicity (yellow), amino acid volume (green) and alanine content (red) at each heptad position of all four silk proteins within their coiled coil region.

The coiled coil domain of the silk structure consists of 24 heptads, forming a continuous rod of 243 Å (Figures 2a,b and S6). The proteins are orientated with F1 and F2 running antiparallel to F3 and F4, with the helices shifted along the superhelical axis relative to each other (axially staggered). For clarity, we denote the chain orientations as up (U) or down (D) based on the direction of the N‐ and C‐terminus along the superhelical axis: F1(U), F3(D), F2(U), and F4(D), arranged sequentially in a clockwise manner. The pitch of the coiled coil is 153 Å, and the radius is approximately 5.8 Å. Within this domain there are six intermolecular electrostatic interactions that potentially link adjacent proteins, except for the F1 and F4, with each interaction located outside the core and within the peripheral heptad positions. The two electrostatic interactions modeled between F2 and F4 (F2_K172_‐F4_D138_ and F4_K145_‐F2_E168_) are present in *c‐c* and *g‐c* positions. The singular electrostatic interaction between F2 and F3 (F2_K214_‐F2_E139_) is between adjacent *b* heptad positions and all three electrostatic interactions between F1 and F3 (F3_R173_‐F1_E100_, F3_R159_‐F1_E114_, and F3_R124_‐F1_E149_) are between adjacent *c* heptad positions (Figure 2d). Analysis of the amino acid composition of each heptad position demonstrated that the positions contributing to the core (*a*, *d*, *e*, and *g*) are the most hydrophobic and have the highest alanine content (Figure 2e). Core positions (*a* and *d*) and those directly adjacent (*g* and *e*) are occupied by relatively small residues compared to the non‐core positions. The core packing is the canonical “knobs‐into‐holes” packing common to the hydrophobic core of many coiled coils, where the side chains of residues at the *a* and *d* positions interlock in alternating layers along the length of the coiled coil (Figure 2c,d). Based on previously published sequence alignments (Campbell et al. 2014; Sutherland et al. 2007), the heptad assignment (*a‐g*) of each protein based on the coiled coil domain are consistent with the heptad positions having been conserved between the protein orthologues (Figure S6).

### 2.3 Hypothesis for the silk coiled coil assembly pathway

**FIGURE 3:**
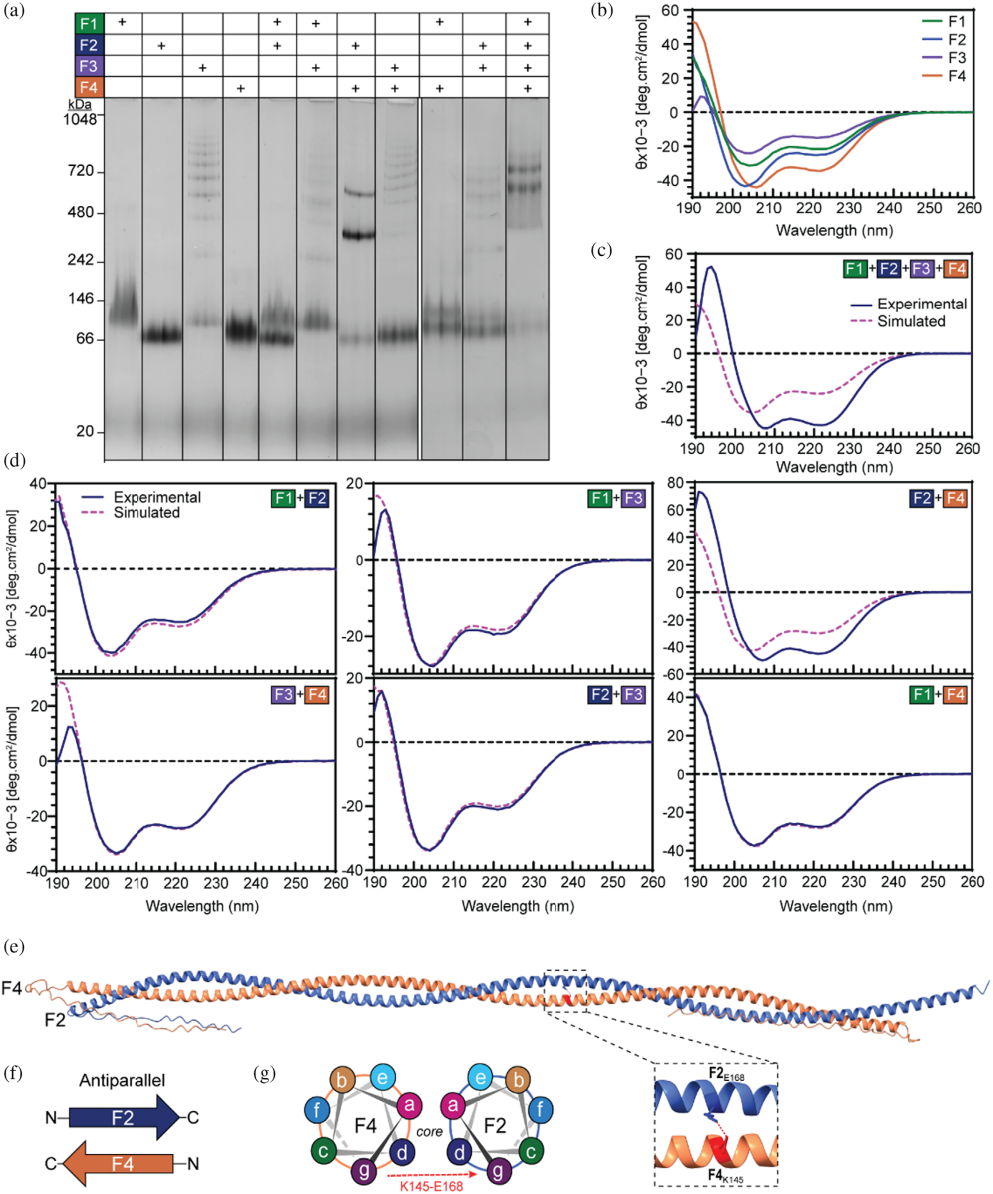
Analysis of initial coiled coil silk formation pathway using BN‐PAGE, CD, and AlphaFold modeling. (a) BN‐PAGE analysis of silk proteins solutions alone and in mixtures. CD spectra of (b) silk proteins alone, (c) in a mixture containing all four proteins or (d) binary mixtures. Data includes experimental spectra (blue) compared to simulated spectra derived from spectra for individual proteins combined (pink dashed). (e) AlphaFold structure of the F2 + F4 dimer. (f) Orientation of F2 and F4 proteins within model. (g) Schematic of a cross‐section of the coiled coil structure showing position of each heptad position (*a‐g*).

The silk proteins F1, F2, and F4 were monomeric in solution, as evidenced by singular bands after BN‐PAGE (Figure 3a). While primarily monomeric, F3 also self‐assembled into larger homomeric species, producing multiple repeating bands larger than the monomer (Figure 3a). CD spectra identified that individual proteins adopted α‐helical structures in solution at the concentrations tested (1 mg/mL), with characteristic minima at 209 and 220 nm, and a ratio of approximately 0.8 (Figures 3b and S7b). When all four proteins were combined, the experimental spectra shifted, retaining the characteristic minima at 209 and 220 nm but with a ratio of >0.9, indicative of coiled coil formation and an increase in the positive maximum at around 192 nm indicating an increase in protein order (Figures 3c and S7c) (Maitip et al. 2015; Zhou et al. 1994). BN‐PAGE analysis of the F1‐F4 sample indicated two distinct bands that migrated above the 480 kDa protein marker (Figures 3a and S7a) supporting the finding that the proteins assembled into higher‐order structures.

We investigated the assembly pathway of the silk tetrameric coiled coil complex using BN‐PAGE and CD spectroscopy, coupled with modeling intermediates in the pathway using AlphaFold. Initially, binary mixtures of the proteins were analyzed to identify significant protein–protein interactions between any two of the silk proteins. No interactions were identified in any of these except in solutions of F2 + F4, where BN‐PAGE identified the formation of a prominent band above the 242 kDa protein marker and a slightly less intense band above the 480 kDa protein marker (Figures 3a and S7a). Similarly, CD analysis of the binary mixtures showed changes in the spectra of the F2 + F4 mixture alone, with an experimental spectrum similar to that of the F1‐F4 mixture indicative of the formation of a coiled coil structure (Figures 3d and S7d). Together, these results suggest that F2 and F4, in the absence of F1 and F3, can interact and form a dimeric coiled coil complex. AlphaFold predicted that F2 and F4 proteins form an antiparallel coiled coil dimer (Figure 3e,f). This structure has an alternating *a* and *d* hydrophobic core and a conserved central ionic interaction within F2 and F4 (F4_K145_‐F2_E168_), which is also present in the final tetrameric structure (Figure 3g). Notably, the heptad positions in the dimer (*a‐g*) in relation to the protein sequence of both F2 and F4 are shifted relative to their positions in the tetramer, with the *a* position in the dimer aligning with the *d* position in the tetramer.

**FIGURE 4:**
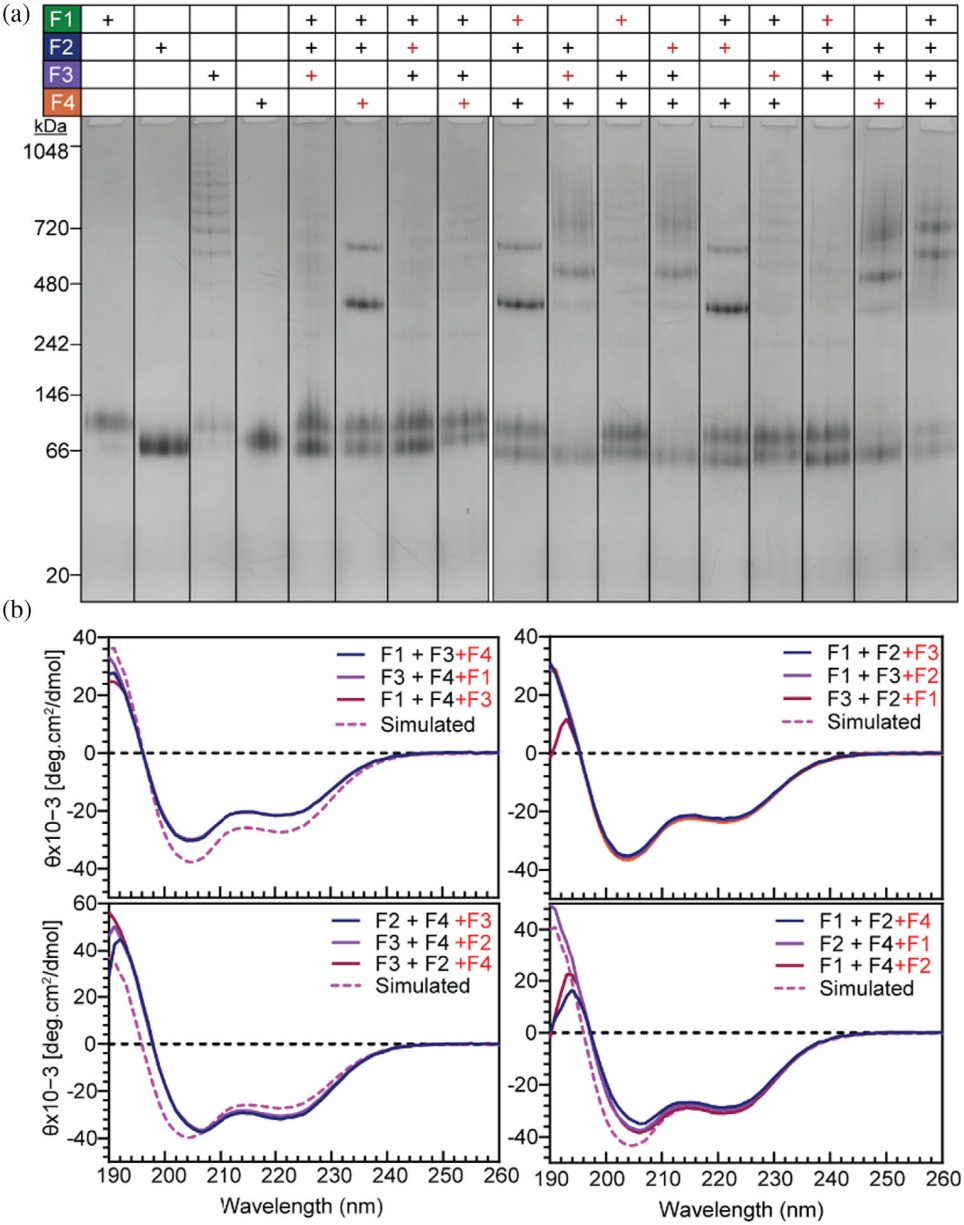
Analysis of intermediate structures during the coiled coil formation pathway using BN‐PAGE and CD. (a) BN‐PAGE analysis of silk protein solutions. Silk proteins indicated with black (+) were initially incubated together before the addition of the final protein indicated with a red (+). (b) CD spectra from tri‐silk protein solutions. Data includes experimental spectra compared to simulated spectra derived from spectra for individual proteins combined (pink dashed).

To investigate intermediate structures in the assembly pathway between the F2 + F4 complex and the final tetrameric structure, ternary mixtures containing three silk proteins in differing combinations were analyzed. Again, no further protein–protein interaction was observed for any mixture (Figures 4a,b and S8a) except when F3 was combined with the F2 + F4 mixture. When F2 + F4 was mixed with F3, a larger complex was formed, as evidenced by the appearance of a distinct, larger band on BN‐PAGE above the 480 kDa protein marker, along with a smeared, less intense higher band (Figure 4a). The CD spectrum of this complex also exhibited the characteristic features of a coiled coil structure (Figures 4b and S8b). Interestingly, for all ternary mixtures, the order in which the proteins were added had no effect on the outcome. The same complexes formed regardless of the sequence of protein addition. Together, these results provide strong evidence for intermediate states in the tetrameric formation pathway. The data support a model in which the F2 + F4 complex acts as a nucleation step, facilitating the formation of intermediate states through the addition of F3, before F1 is incorporated to complete the heterotetrameric silk coiled coil complex.

### 2.4 Probing the dynamics of the silk tetrameric coiled coil assembly pathway

**FIGURE 5:**
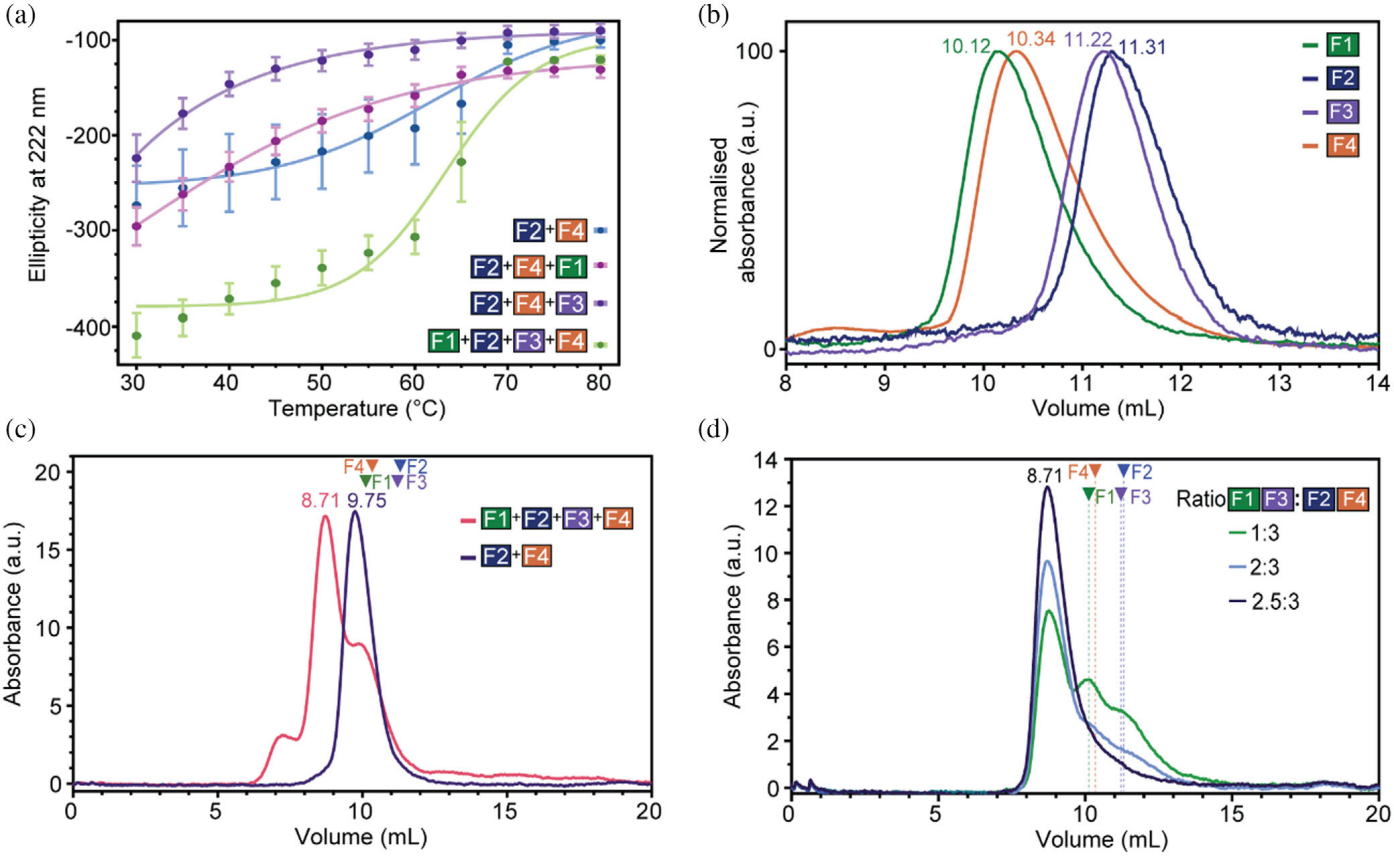
Validation of assembly process using thermal melting CD and analytical size exclusion chromatography. (a) Melting curves of silk protein mixtures monitored by CD signal at 222 nm reported as mean ± standard deviation (*n* = 3). Analytical size exclusion chromatography profiles of silk proteins (b) alone and (c) bi‐ and tetrameric mixtures of the silk proteins, with the latter containing 20% excess of F2 & F4. (d) SEC profiles of mixtures containing the four silk proteins at varying ratios of F2/F4 to F1/F3. All samples had a total concentration of 4 mg/mL. Elution volumes of the individual silk proteins are indicated on the chromatograms as colored triangles and dotted lines.

We analyzed the stability and dynamics of the species that form along the silk coiled coil assembly pathway. The thermal stability of these species was probed by CD (Figures 5a and S9). Comparisons of the protein mixtures measured at the same concentrations showed that the F1‐F4 complex exhibited the lowest initial ellipticity at 222 nm compared to other mixtures, suggesting the four proteins form a highly ordered complex at lower temperatures (30–40°C). The curve displayed a clear sigmoidal transition with a *T*_*m*_ at approximately 63°C, indicating the four proteins unfolded via a cooperative process. Similarly, the thermal melting curve of the F2 + F4 mixture was also sigmoidal in shape (*T*_*m*_ ≈ 61°C), suggesting the dimer also unfolds in a cooperative manner. However, this mixture showed a higher initial ellipticity and smaller change with increasing temperature compared to the F1‐F4 mixture, suggesting the dimer is less structured compared to the tetramer. Addition of F1 or F3 to F2 + F4 altered the shape of the melting curves, suggesting these mixtures have a non‐cooperative unfolding process. Together, these findings support a stepwise assembly model where F2 and F4 form a stable core, F3 then binds this complex, and lastly F1 initiates formation of the final, highly ordered structure.

This assembly pathway implies that the amount of tetramer formed will be influenced by the relative ratios of F1 and F3 to F2 + F4. We quantified the relative amount of tetramer formed with different protein ratios using size exclusion chromatography (SEC). Mixtures of all four proteins (F1‐F4) containing a 20% excess of F2 and F4 produced peaks consistent with equimolar mixtures of F1‐F4 relative to the added F1 and F3 (a small peak at 7.10 mL and a dominant peak at 8.71 mL), plus a smaller peak at 9.75 mL consistent with the elution profile of the F2 + F4 dimer (Figure 5c). Under these conditions, no peaks corresponding to individual proteins were observed (F1: 10.12 mL; F2: 10.34 mL; F3: 11.22 mL; F4: 11.31 mL) (Figure 5b) consistent with all proteins being present within coiled coil complexes of F1‐F4 or F2 + F4. Analysis of mixtures containing all four proteins (F1‐F4) with increasing deficits of F2 and F4 produced decreases in the dominant tetramer peak (8.71 mL) and increases in the peaks corresponding to F1 (10.12 mL) and F3 (11.22 mL) (Figure 5d). No peaks corresponding to the dimer (F2 + F4) or F2 or F4 monomers were observed. These results emphasize the critical role that the F2 + F4 complex plays in facilitating the transition to the fully assembled heterotetrameric coiled coil structure.

## 3 DISCUSSION

Our AlphaFold modeling, crosslinking analysis, and cryo‐EM structure confirm that the four silk proteins form a heterotetrameric coiled coil structure. This finding aligns with X‐ray diffraction data from silk materials (Atkins 1967; Kameda and Tamada 2009; Rudall 1962; Weisman et al. 2010) and agrees with evolutionary biology and biochemical data suggesting that each silk protein ortholog has a distinct, non‐redundant function in the silk (Maitip et al. 2015; Sutherland et al. 2007, 2012). Since the four silk proteins arose from gene duplication events, the heptad periodicity within each ortholog would be expected to be under evolutionary constraint (Sutherland et al. 2006). Comparisons of ortholog sequence alignments (Campbell et al. 2014; Sutherland et al. 2006) and the heptad periodicities observed in our structure (Figure S6) find that the periodicity is retained between proteins, further validating the accuracy of the model.

Within the coiled coil, the four proteins adopt an antiparallel configuration with each protein having a two‐residue core (*a‐d*) and knobs‐in‐holes packing. This packing is distinct from the hypothesized “Alacoil” model, which features a three‐residue core (*x‐da*) (Sutherland et al. 2006). The coiled coil length in the model is 24.3 nm. Bioinformatic predictions for the individual silk proteins AmelF1‐F4 indicate the potential for even longer coiled coil lengths of 38, 41, 36, and 36 nm, respectively (Fraser and Parry 2015). The AlphaFold model does not support the entire length of the coiled coil region identified in the bioinformatic analysis or previous experimental data. The shorter length of the coiled coil observed in our model, with relatively unstructured extremities, is probably a result of a weaker coevolutionary signal among the orthologous silk proteins in these coiled coil extremities, resulting in the lower confidence, less regular structural predictions by Alphafold in these regions (Figure S2).

The N‐ and C‐terminal regions of the silk proteins were predicted to be unstructured with low structural prediction scores. These regions of the proteins across species have diverged extensively, both in sequence length and amino acid composition (Campbell et al. 2014; Kameda and Tamada 2009; Sutherland et al. 2014). In the hornet silks, these regions have been shown to be associated with β‐sheet structure thought to cross‐link the coiled coils into the final material (Kameda 2012) and are believed to play a similar role in other silks. As the β‐sheet cross‐linking does not require highly sequence‐specific protein‐to‐protein interactions, any coevolutionary signal between the proteins would be weaker resulting in a lower confidence and potentially less accurate AlphaFold structure prediction for these regions.

The radius of the honeybee coiled coil is 5.8 Å, which is larger than the 5.2 Å proposed by Atkins (1967). However, Atkins conceded that the value he proposed could be increased while still maintaining good agreement with the data (Atkins 1967). Both our model and Atkin's X‐ray diffraction data indicate relatively small radii compared to the typical radii of tetrameric coiled coils (6.8–8.0 Å) (Deng et al. 2006). This is likely the consequence of small amino acids (such as alanine) being present within the core positions of the bee silk coiled coil (Figure 2e) compared to larger amino acids such as leucine, valine, and isoleucine found commonly at core positions in other coiled coils (Lupas et al. 2017). Alanine's small side chains enable tighter packing of the helices; for example, when alanine is substituted for larger hydrophobic residues (leucine, valine, and isoleucine) into the core of the *E. coli* Lpp‐56 trimeric coiled coil, the radius is reduced from 6.1 to 5.2 Å (Liu and Lu 2002). Given that pitch depends on superhelical radius (Lupas and Gruber 2005), the small radius of bee silk coiled coil explains the shorter pitch observed in our structure (153 Å), compared to the typical pitch for a tetrameric coiled coil (around 188 Å) (Lupas and Gruber 2005). Experimental data measuring pitch from solid state silk materials are inconclusive, with Atkins (1967) measuring a pitch of 140 ± 4 Å in honeybee silk, derived from a near equatorial layer line spacing of 3.5 ± 0.1 nm in the X‐ray diffraction pattern, and Kameda et al. (2014) measured a pitch of 172 Å for the giant hornet (*Vespa mandarinia japonica*) silk.

Convention suggests that interhelical electrostatic interactions between *i* to *i* + 5 from heptad position *g* to *e*/*g* controls the axial positioning and oligomerization of helices within coiled coils. In the silk coiled coil structure, we identified six potential intermolecular electrostatic interactions, none of which were *e‐e* or *g‐g* interactions. Instead, the electrostatic interactions were located between *c‐c*, *g‐c*, and *b‐b*. Antiparallel tetramers bring residues in the *c* and *b* position into close enough proximity to their respective counterparts to allow electrostatic interactions between *c‐c* and *b‐b* positions (*i* to *i* + 6) (Meier et al. 2010). In fact, analysis of known antiparallel tetrameric coiled coils has showed that the classic *i* to *i* + 5 interactions are not the most frequently observed electrostatic interactions and that various other salt bridges are observed (Meier et al. 2010). In the honeybee silk, we propose that the small size of the residues in the core and *g* positions, combined with the antiparallel tetrameric structure brings the *c* and *b* residues into proximity with the equivalent residues on neighboring strands to allow preferential electrostatic interactions.

**FIGURE 6:**
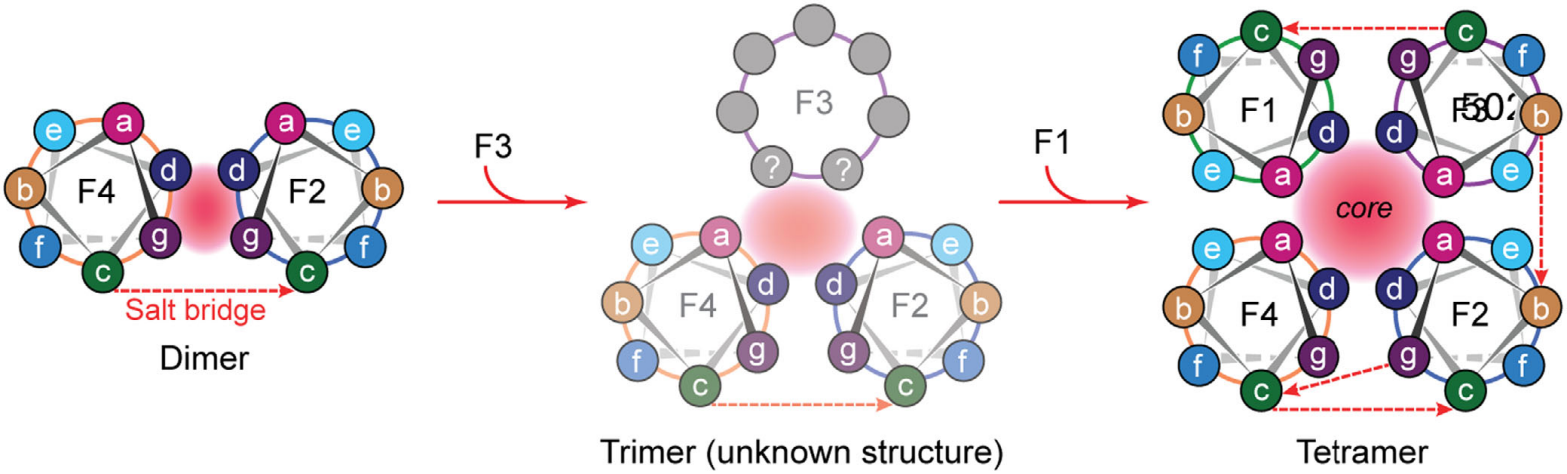
Proposed assembly pathway of the silk tetramer. Heptad positions assigned based on heptad assignments of proteins in the tetrameric structure. The red areas indicate the predicted core within each structure. Dotted red lines show the positions of the ionic interactions (salt bridges).

The conventionally accepted mechanism for coiled coil formation is a two‐state transition from unfolded monomers to the coiled coil structure, initiated by trigger sequences that form a nucleation site, which promotes correct chain‐to‐chain association (Lupas and Gruber 2005). While this mechanism has been demonstrated multiple times for dimeric coiled coils, few investigations have explored into the mechanistic details of the folding pathway of natural tetrameric coiled coils. Here we propose that the heterotetrameric coiled coils of the honeybee silk proceed through a four‐state transition pathway involving α‐helical proteins that assemble sequentially into dimer and trimeric intermediates, leading to the final tetrameric structure (Figure 6). Using CD and BN‐PAGE, we observe species consistent with stable intermediate structures that support this model. In our proposed pathway, F2 and F4 first fold into a dimer, with the eventual *g* and *d* heptad positions (defined based on the tetramer) forming the hydrophobic core, which is stabilized with electrostatic interactions. F3 then associates with the dimer forming a structure that contains a coiled coil but is yet to be determined either empirically or predicted computationally. Finally, the addition of F1 leads to folding of the heterotetramer, a slight rotation of the helices so that *a* and *d* heptad positions form the core shifting the principal heptad register to that which is found in the tetramer, and where the entire structure is locked in place with additional electrostatic interactions and hydrogen bonding, hydrophobic interactions and complementary sterics through knobs‐into‐holes fitting.

Bioinformatic analysis using MARCOIL indicates that all the silk proteins have offset heptad repeats throughout their predicted coiled coil domain. Heptad offsets generate multiple hydrophobic faces along the alpha helix and have been related to coiled coil oligomer state (Walshaw and Woolfson 2001). Notably, previously reported heptad assignments for the silk proteins based on the dominant MARCOIL heptad prediction (Campbell et al. 2014; Sutherland et al. 2007) are correct for the F2 + F4 dimer intermediate but do not align with the final tetrameric structure. We propose that the dimer is built on one heptad offset, while the final structure adopts a second heptad register which is also predicted by MARCOIL (Figure S6). In the context of the final tetramer, the helices adopt a bifaceted interaction mode, with two distinct sets of interfaces between subunits. We propose that this transition between heptad registers, combined with the high abundance of alanine at the *d* and *g* positions in the tetramer, facilitates the initial dimerization of F2 + F4 and the subsequent assembly of the tetramer. Similarly, a high abundance of alanine at the *g* positions is associated with the formation of the higher‐order oligomeric states in other systems (Deng et al. 2007; Liu et al. 2007).

The proposed assembly process of the honeybee coiled coil represents a unique pathway that facilitates the proper formation of the tetrameric building blocks essential for silk material assembly. We believe this process, the first described for a natural heterotetrameric coiled coil, plays a key role in ensuring the proper assembly of the coiled coils, thereby facilitating their ability to pack into ordered liquid crystals and, ultimately, the final silk material. Importantly, given the interest in utilizing these silk materials for de novo design, resolving the tetrameric sequence–structure relationship provides critical information necessary for the rational design of novel functionalities into these silk proteins. These insights pave the way for the development of innovative silk‐based technologies with tailored functionalities for a diverse range of applications.

## 4 MATERIALS AND METHODS

### 4.1 Recombinant silk protein expression and purification

Recombinant expression constructs for honeybee silk genes from *Apis* species that have been previously used to express silk proteins at high levels in *E. coli* were used in this study (Shilling et al. 2025). Constructs for three genes FJ235089, FJ235090, and FJ235091 (GenBank accession numbers) were described in Weisman et al. (2010). The fourth silk protein ortholog gene (KC708023) was codon optimized for *E. coli* and synthesized in pET9a, as described in Shilling et al. (2025). Silk proteins were expressed as previously described (Weisman et al. 2010).

For silk protein purification, cells were incubated for 20 min at room temperature in lysis buffer (100 mM Tris–HCl, pH 7.0; 5 mM EDTA; 5 mM DTT; 5 mM benzamidine HCL; 200 μg/mL lysozyme) before undergoing lysis by sonication (full power, 50% duty cycle and 5 s pulses) and high‐pressure homogenization (20 K psi; Avestin Emulsiflex C3). Inclusion bodies (IBs) were collected by centrifugation and washed three times with wash buffer (100 mM Tris HCl, pH 7.0; 5 mM EDTA; 5 mM DTT; 2M urea; 2% (w/v) Triton X‐100), followed by washes with and without urea or Triton X‐100 and a final wash with 1M guanidinium. IBs were solubilized in 8M guanidinium, clarified by centrifugation, and dialyzed overnight (10,000 MWCO) against ×100 volume of water overnight at 4°C. Insoluble matter was removed by centrifugation.

Silk proteins were purified by weak anion exchange chromatography using Fractogel® EMD DEAE resin (Merck), diluted at least 2‐fold with 20 mM Tris–HCl, pH 7.15, incubated with shaking for 30 min, and loaded onto a gravity column (5–6 cm packed bed). Proteins were eluted in 20 mM Tris, pH 6.8, 100–200 mM NaCl, sterile‐filtered (0.22 μm), quantified by BCA protein assay (Pierce) and stored at −80°C.

### 4.2 AlphaFold structural prediction of silk protein complexes and model analysis

Honeybee silk protein sequences (derived from GenBank accession numbers: FJ235089, FJ235090, FJ235091, KC708023) were used to generate five predicted tetrameric structures with AlphaFold2 (v2.3.2; Jumper et al. 2021). The highest‐ranking model was used for all analysis. It exhibited high pLDDT scores (70–90) in the central coiled coil region, while the terminal regions had lower confidence scores (pLDDT <70) (Figure S2a). AlphaFold3 (Abramson et al. 2024) predicted a similar coiled coil structure, but it was excluded due to steric clashes between amino acid side chains within the coiled coil region that were not present in the AlphaFold2 models (Table S1). A dimeric F2 and F4 structure generated with AlphaFold3 displayed high pLDDT scores (>70) within the coiled coil region, with lower confidence scores in the terminal regions (pLDDT <70) (Figure S2b). Predicted models were analyzed in ChimeraX (Meng et al. 2023) to assess relative protein orientation, heptad repeat positions (*a‐g*), core packing, and ionic interactions. In the predicted tetrameric coiled coil model, heptad positions were assigned and used to calculate the average hydrophobicity, amino acid volume, and alanine content for each position across all four proteins. Hydrophobicity values were assigned according to the Kyte and Doolittle scale (Kyte and Doolittle 1982).

### 4.3 Cross‐linking and mass spectrometry

Silk proteins (F1‐F4) were combined in equal ratios at 0.2 or 2 mg/mL in 100 mM sodium phosphate, pH 7.2, or 50 mM ammonium bicarbonate, pH 8, respectively. BS^3^ crosslinking was conducted according to the manufacturer's instructions (ThermoScientific) and visualized on 4–12% Bis‐Tris polyacrylamide gels (Invitrogen). Excised bands were incubated in 50 mM ammonium bicarbonate for 10 min at room temperature. The solution phase was removed and 0.25 μg of sequencing grade trypsin (Promega) in 50 μL 50 mM ammonium bicarbonate was added and incubated at 37°C for 3 h. The digestion was stopped with 1 μL of 10% (v/v) formic acid and filtered with a 0.22‐μm filter.

Tryptic peptides were desalted and concentrated with a trap column (PepMap100 C18, 5 mm × 300 μm, 5 μm, ThermoScientific) and separated on a nano column (PepMap100 C18, 150 mm × 75 μm, 2 μm, ThermoScientific) using an UltimateTM 3000 RSLC nano LC system (ThermoScientific). Mobile phase A consisted of water and 0.1% (v/v) formic acid, and mobile phase B consisted of 80% (v/v) acetonitrile and 0.08% (v/v) formic acid. Tryptic peptides were eluted using a gradient of 5%–60% solvent B for 20 min and 60%–99% solvent B for 5 min. The eluted peptides were ionized with a Nanospray Flex Ion Source (ThermoScientific) with spray voltage set to 2.3 kV and the heated capillary at 300°C. Mass spectra (MS) and tandem MS/MS analysis were performed using an Orbitrap Fusion MS (ThermoScientific). MS survey scans of peptide precursors were performed in the Orbitrap detector with scan range of 400–1500 *m*/*z*, at resolution of 120 K (at 200 *m*/*z*). The target value of automatic gain control was set as 4 × 10^5^. The maximum injection time for the MS was 50 ms. The most abundant precursors of charge states 2+ to 7+ with intensity greater than 1 × 10^5^ were isolated by the quadrupole with a window of 1.6 *m*/*z*. MS/MS fragmentation was achieved by high‐energy collisional dissociation with collision energy of 28%. Fragments were detected in the ion trap detector in rapid scan rate mode.

Spectrum data files were analyzed using Protein Discoverer 3.1 software (ThermoScientific) using the Xlink node for crosslinked peptides and Sequest HT search engine for unmodified and dead‐end‐modified peptides. Peptide spectral matches were validated using the Percolar algorithm, based on *q*‐values and 1% false discovery rate. Precursor mass tolerance was set to 10 ppm, and product ions were searched at 0.02 Da. Three missed tryptic cleavages were allowed. Modifications included BS^3^ crosslinked mass modification (+138.068 Da) and oxidation (+15.995 Da).

### 4.4 Cryogenic electron microscopy sample preparation

The four silk proteins were combined in equimolar amounts and concentrated to 10 mg/mL using 10,000 MWCO Vivaspin® 20 concentrators (Sartorius). The mixture was loaded onto a Superdex200 HiLoad 16/600 size exclusion column (Cytiva) pre‐equilibrated in 20 mM Tris–HCl, pH 7.0. Eluted fractions were collected and analyzed by blue native polyacrylamide gel electrophoresis (BN PAGE) (Invitrogen). Fractions containing the tetrameric silk protein complex were combined, concentrated to 5 mg/mL, and stored at −80°C until further use. For subsequent analysis, the frozen sample was thawed and diluted in 20 mM Tris–HCl, 100 mM NaCl, pH 7.4, to appropriate concentrations for initial screening using negative stain transmission electron microscopy (TEM) and cryo‐EM. For TEM screening, 3 μL of the sample (0.025 mg/mL) was applied to a glow‐discharged, standard Formvar on carbon copper TEM grid (ProSciTech, GSFC100CU) for 2 min, blotted, washed with water, stained with 2% uranyl acetate solution for 2 min, and air‐dried. TEM images were collected on a JEM‐F200 (JEOL) transmission electron microscope equipped with a Gatan Rio16 CMOS camera. For cryo‐grid preparation, 3 μL of the sample (2.5 mg/mL) was applied to a glow‐discharged UltrAUFoil grid (R 1.2/1.3). The grid was blotted for 2 s at 5°C with 100% humidity and plunge‐frozen in liquid ethane using a Vitrobot Mark IV (ThermoScientific).

### 4.5 Cryo‐EM data acquisition and processing

Data acquisition was performed on a CRYO ARM 200 (JEOL) electron microscope equipped with an in‐column Omega energy filter and DE64 detector. Several data sets (*n* = 3) were collected using SerialEM software (Mastronarde 2005) under counting mode at a magnification of 120,000× (pixel size 0.996 Å) with a defocus range of −0.5 to −2.0 μm. The combined data sets had 3496 movies and each movie consisted of 32 frames recorded with a dose rate of 40e^−^/Å^2^s^−1^. The acquired data was processed using cryoSPARC‐4.5.3 (Punjani et al. 2017). The micrographs from each movie were aligned using Patch Motion Correction and Patch contrast transfer function (CTF) estimation. A total of 121,180 particles were picked and passed through multiple rounds of two‐dimensional (2D) classification and class selection, with the best 38,205 particles selected for ab initio three‐dimensional (3D) reconstruction. The predicted silk tetrameric structure generated with AlphaFold2 was fitted into the density map in ChimeraX (Meng et al. 2023).

### 4.6 Circular dichroism spectroscopy

Silk proteins (1 mg/mL) or mixtures, in 20 mM Tris–HCl, pH 7.4, 100 mM NaCl, were incubated on ice for 30 min before measurement using a 0.2 mm path‐length cell (Starna, 20/0/Q/0.2) in a Chirascan CD spectrometer. Tri‐protein mixtures were prepared by the addition of a third silk protein stock solution to the bi‐protein solutions just prior to measurement via circular dichroism (CD) resulting in each protein being present at 0.5 mg/mL. Spectra were collected at 20°C (190–260 nm, 1 mm bandwidth, 120 nm/min scan rate) and buffer‐corrected. All samples were measured 3 times, and the average was reported.

Thermal denaturation CD spectra were measured on 0.1 mg/mL silk protein mixtures in 20 mM Tris–HCl, pH 7.4, 100 mM NaCl, over a temperature range of 30–80°C with heating steps of 1°C/min. The change of ellipticity at 222 nm was monitored over the temperature range in an increment of 5°C and reported as mean ± standard deviation (*n* = 3) of three independent experiments.

### 4.7 Blue native polyacrylamide gel electrophoresis

BN‐PAGE was performed using NuPAGE Bis‐Tris 4–12% polyacrylamide gel (as per manufactures instructions, ThermoScientific) with 0.1 mg/mL final concentration protein mixtures in 20 mM Tris–HCl, pH 7.4, 100 mM NaCl, and NativeMARK Unstained Protein Standards (ThermoScientific).

### 4.8 Analytical size exclusion chromatography

Silk proteins (4 mg/mL) in 20 mM Tris, pH 7.4, 100 mM NaCl were spun at 20,000*g* for 20 min to remove any insoluble protein, then loaded (100 μL) onto a Superdex200 10/300 GL size exclusion column (GE Healthcare) equilibrated with the same buffer. Proteins were eluted at 0.3 mL/min, and an in‐line UV detector (280 nm) was used to monitor protein elution.

## AUTHOR CONTRIBUTIONS

**Caitlin L. Johnston:** Conceptualization; data curation; investigation; methodology; project administration; validation; visualization; writing – review and editing; writing – original draft; formal analysis. **Chacko Jobichen:** Data curation; investigation; resources; formal analysis. **Lyndall J. Briggs:** Investigation; writing – review and editing. **Michelle Michie:** Writing – review and editing; resources. **Jian‐Wei Liu:** Investigation; data curation; formal analysis; writing – review and editing. **Craig J. Morton:** Funding acquisition; conceptualization; supervision; writing – review and editing. **Andrew C. Warden:** Funding acquisition; conceptualization; resources; supervision; writing – review and editing. **Tara D. Sutherland:** Funding acquisition; conceptualization; methodology; project administration; resources; supervision; writing – original draft; writing – review and editing.

## CONFLICT OF INTEREST STATEMENT

The authors declare no conflicts of interest.

## Supporting information


**Data S1.** Supporting Information Figures 1–9.

**Data S2.** Xlink—Crosslinking raw data.

**Data S3.** Supporting Information.

## Data Availability

The cryo‐EM map is available in the EM Data Bank (EMDB) with accession number EMD‐49474. The authors declare that all other data supporting the findings are available within this paper and its Supporting Information files.
