# Supplementary material for: Resolving the structure and assembly of the honeybee silk heterotetrameric coiled coil
Source: Protein Sci. 2025 Jul 23;34(8):e70230. doi: 10.1002/pro.70230 (PMC12284834; doi:10.1002/pro.70230)
Supplement: Supplementary file 1 — Data S1. Supporting Information Figures 1–9. [file PRO-34-e70230-s001.docx]

**Supplementary information**

**Resolving the structure and assembly of the honeybee silk heterotetrametric coiled coil**

Caitlin L Johnston^a*^, Chacko Jobichen^b,e^, Lyndall J. Briggs^a^, Michelle Michie^a^, Jian-Wei Liu^c^, Craig J. Morton^d^, Andrew C. Warden^c^, Tara D. Sutherland^a^

^a^ Health & Biosecurity, CSIRO, Canberra, ACT 2601, Australia

^b^ Centre for Advanced Microscopy, Australian National University, Canberra, ACT, 2601, Australia

^c^ Environment, CSIRO, Canberra, ACT, 2601, Australia

^d^ Manufacturing, CSIRO, Parkville, VIC, 3052, Australia

^e^ Present address: School of Chemistry and Molecular Biosciences, The University of Queensland, QLD, 4072, Australia

**Corresponding Author:**

Caitlin L. Johnston, CSIRO Health & Biosecurity, Canberra, ACT 2601, Australia; [caitlin.johnston@csiro.au](mailto:caitlin.johnston@csiro.au)

This file includes:

Supplementary Figures 1 to 9

**Supplementary Figures**


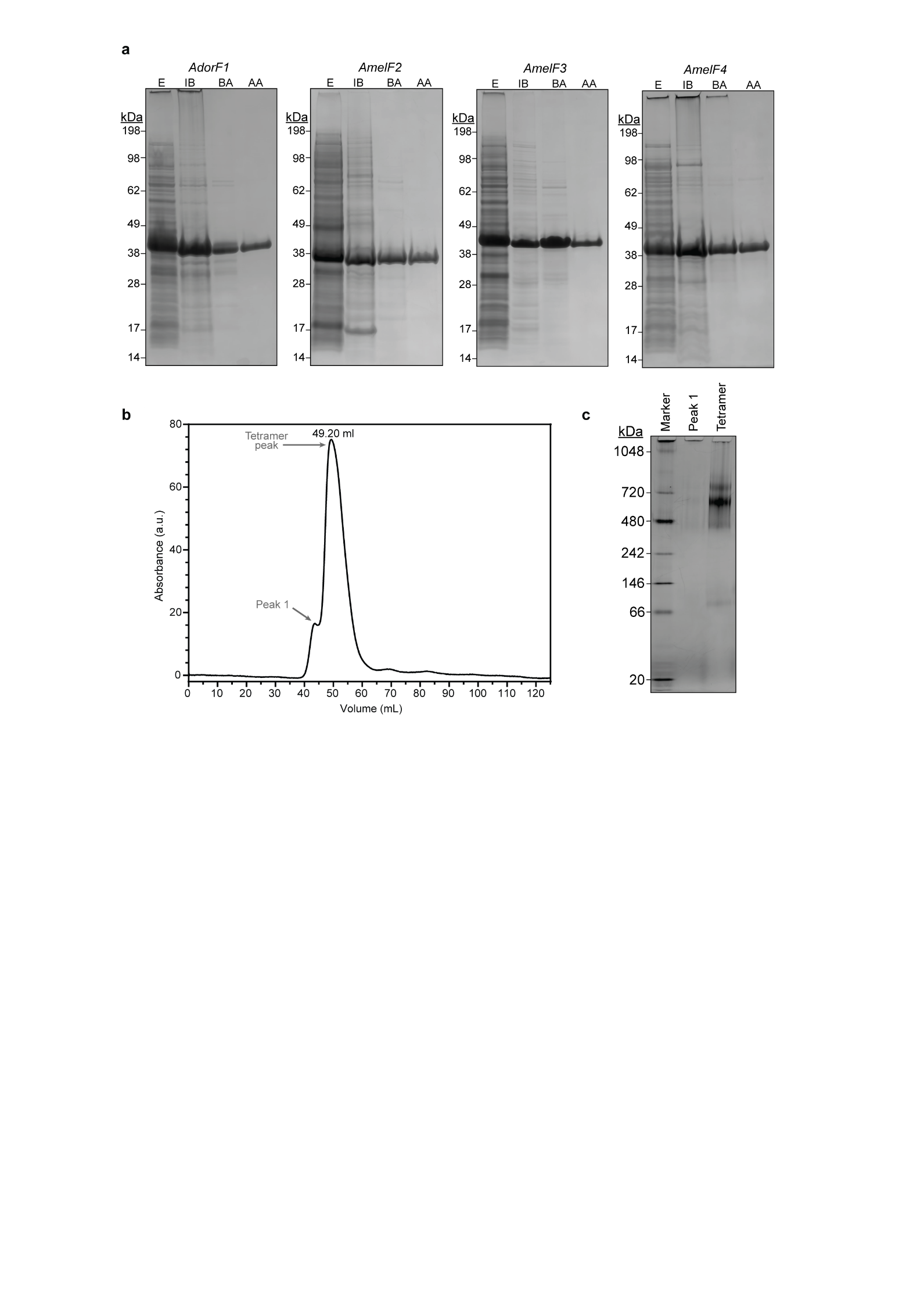


**Supplementary Fig.1:** **Purification of silk proteins.** (a) SDS-PAGE analysis of protein purification steps of silk proteins from *E. coli* expressions. Each gel shows the expression fractions (E), inclusion body pellet (IC) and the protein before (BA) and after (AA) anion exchange chromatography. (b) Size exclusion chromatogram of purification of silk protein tetramer for Cryo-EM. (c) BN-PAGE analysis of SEC peaks.


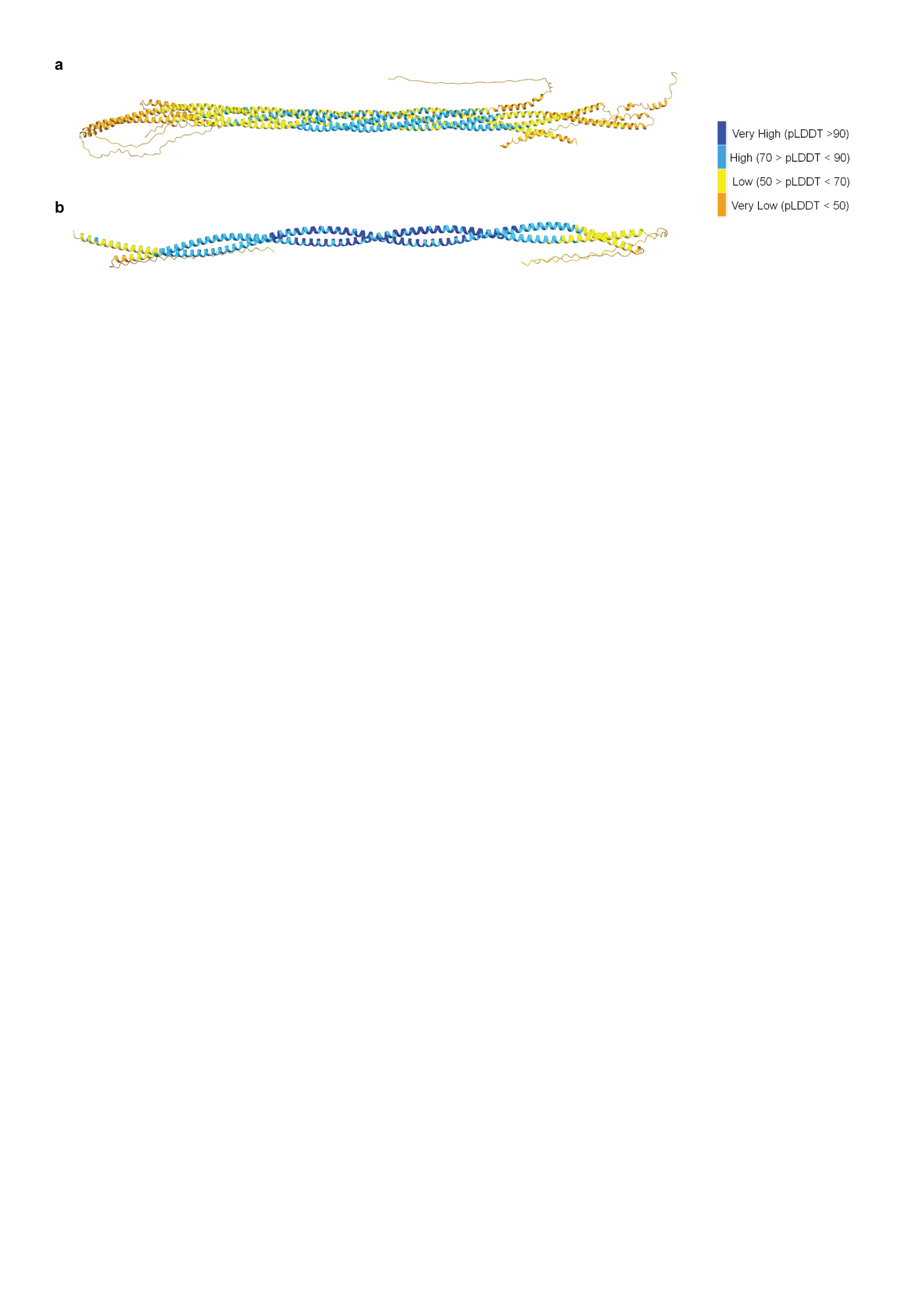


**Supplementary Fig.2:** pLDDT scores of predicted protein models generated using (a) AlphaFold2 for the silk tetramer (F1-F4) and (b) AlphaFold3 for the silk dimer (F2 and F4). Models shown in cartoon and coloured according to pLDDT scored from 0 to 100.

**Supplementary Table 1:** MolProbity summary statistics for analysis of all-atom contacts and geometry for silk tetrameric models generated using AlphaFold2 and AlphaFold3 (Williams et al., 2018).

|  |  | **AlphaFold2 model** | | **AlphaFold3 model** | |  |
| --- | --- | --- | --- | --- | --- | --- |
| All-Atom Contacts | **Clashscore, all atoms:** | **0** | **100^th*^ percentile** | **22.58** | **26^th^* percentile** | (N= 1784, all resolutions) |
|  | Clashscore is the number of serious steric overlaps (> 0.4 Å) per 1000 atoms | | | | | |
| Protein Geometry | Poor rotamers | 13 | 1.65% | 14 | 1.78% | Goal: <0.3% |
|  | Favored rotamers | 722 | 91.74% | 746 | 94.79% | Goal: >98% |
|  | Ramachandran outliers | 3 | 0.24% | 28 | 2.28% | Goal: >0.05% |
|  | Ramachandran favored | 1179 | 96.24% | 1153 | 93.82% | Goal: >98% |
|  | Rama distribution Z-score | -4.20 ± 0.17 | | 3.75 ± 0.20 | | Goal: abs(Z-score) < 2 |
|  | **MolProbity scoreˆ** | **0.92** | **100^th^ percentile** | **2.45** | **50^th^ percentile** | (N=27675, 0Å - 99Å) |
|  | Cβ deviations >0.25Å | 83 | 6.96% | 0 | 0.00% | Goal: 0 |
|  | Bad bonds | 182/8639 | 2.11% | 24/8645 | 0.28% | Goal: 0% |
|  | Bad angles | 525/11685 | 4.49% | 29/11685 | 0.25% | Goal: <0.1% |
| Peptide Omegas | Cis Prolines | 0/11 | 0.00% | 0/11 | 0.00% | Expected: <1 per chain. |
|  | Cis nonProlines | 15/1220 | 1.23% |  |  |  |
|  | Twisted Peptides | 4/1231 | 0.32% | 2/1233 | 0.16% | Goal: 0 |
| Low-resolution Criteria | CaBALM outliers | 21 | 1.7% | 2 | 0.2% | Goal: <0.1% |
|  | CA Geometry outliers | 12 | 1.07% | 0 | 0.00% | Goal: <0.5% |

In the two column results, the left column gives the raw count, right column gives the percentage.

* 100^th^ percentile is the best among structure of comparable resolution; 0^th^ percentile is the worsts. For clashscore the comparative set pf structures was selected in 2004, for MolProbity score in 2006.

ˆ MolProbity score combines the clashcore, rotomer, and the Ramachandran evalutions into a single score, normalized to be on the same scale as X-ray resolution.


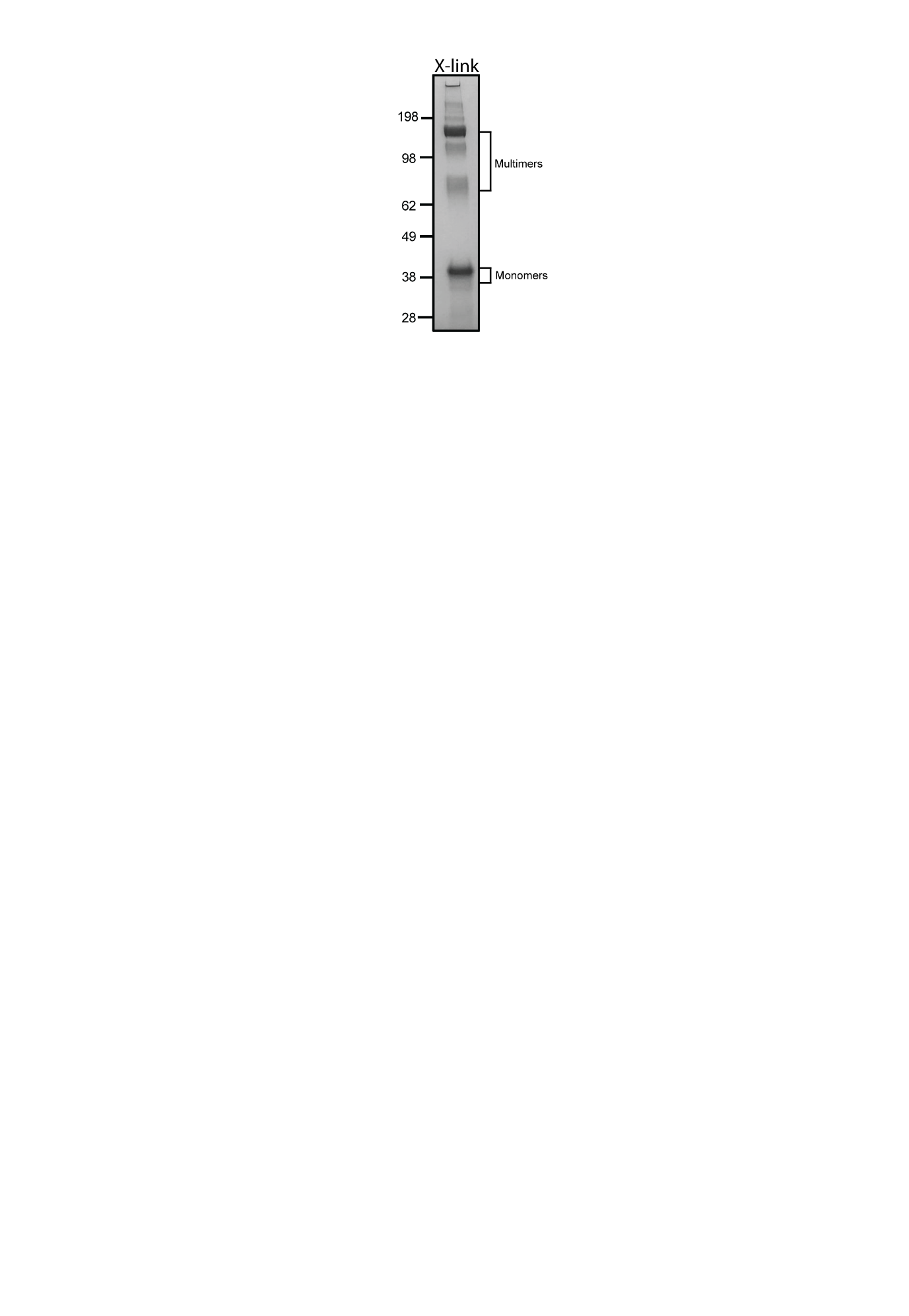


**Supplementary Fig.3:** SDS-PAGE gel of F1-F4 protein mixture at 0.2 mg/mL crosslinked (X-link) with BS^3^ crosslinker.


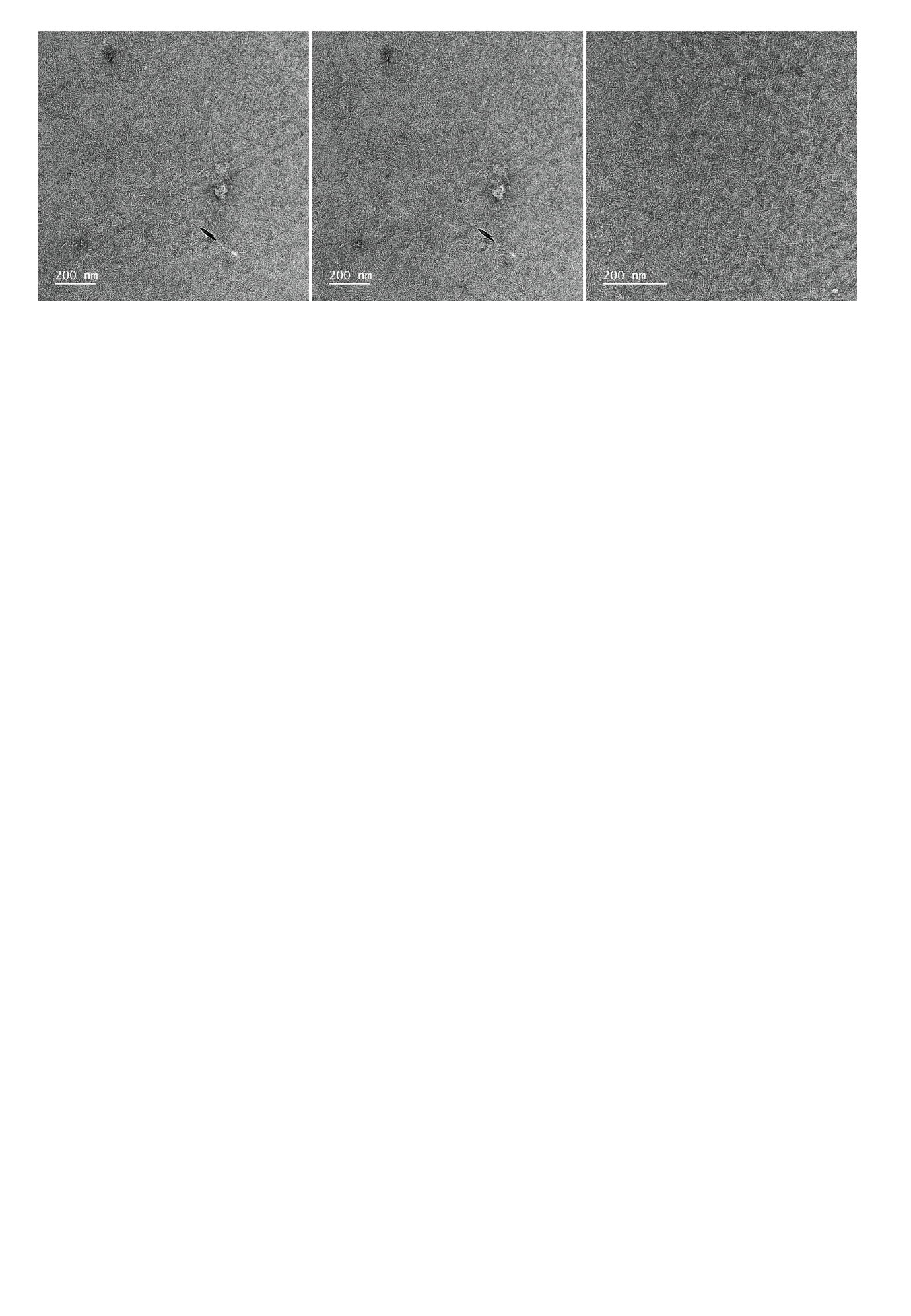


**Supplementary Fig.4:** Example transmission electron microscopy images from initial screening of F1-F4 mixtures showing the large number of particles present.


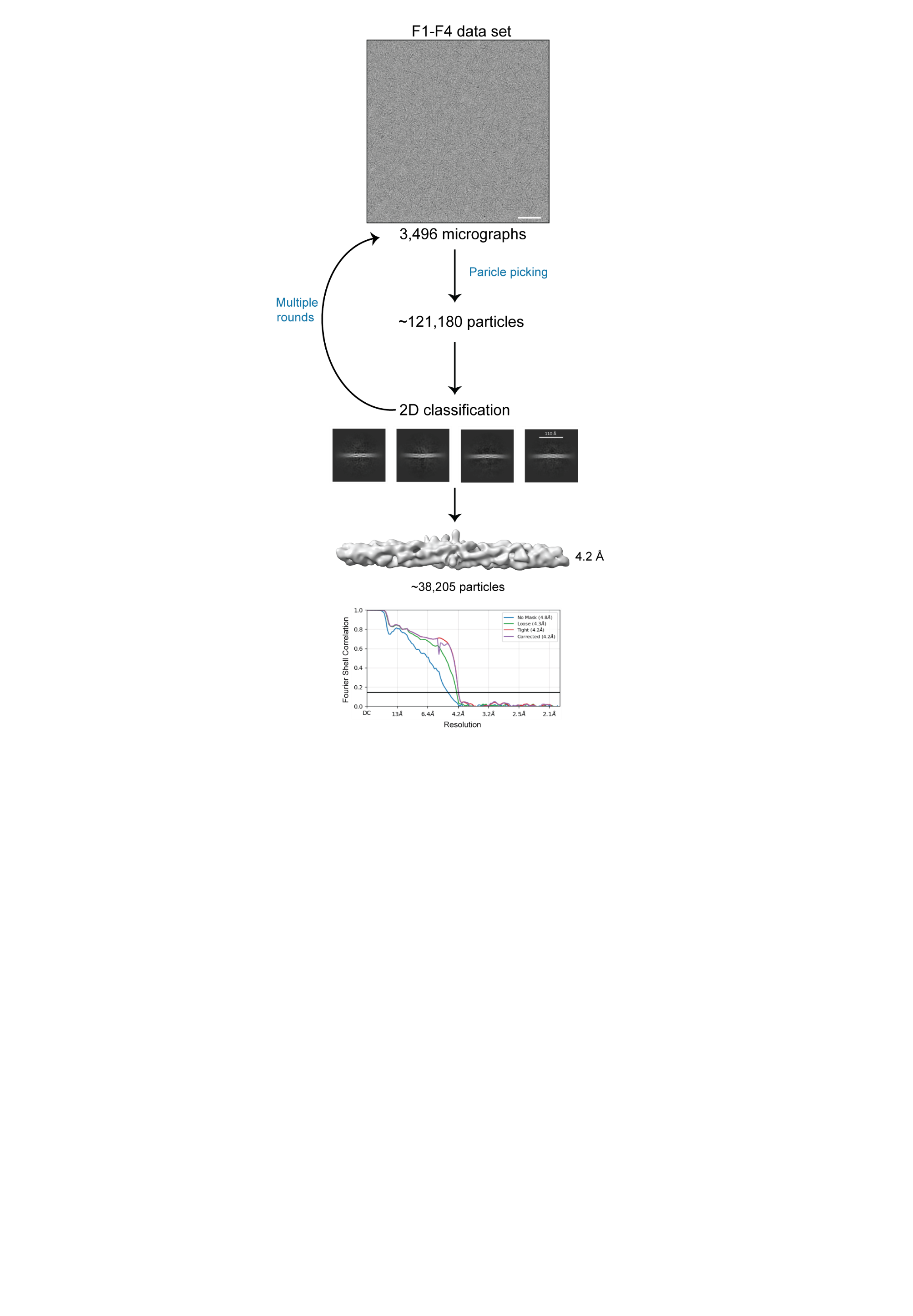


**Supplementary Fig.5:** **Cryo-EM structure determination of F1-F4 coiled coil complex.** Representative micrograph of F1-F4 particles and workflow of data processing, showing representative 2D classes, density map and FSC curves for the complex.

Heptad *abcdefgabcdefgabcdefgabcdefgabcdefgabcdefgabcdefga*

F1 12 VKGSASATASTAVTARSGLRAGQV**ALASQKDAVLQAEAAASAASEARAAA** 62

F2 32 SIENAQKVARAAENVGLNLELGAG**ARAASVAAAAQAKNTEAAEAGANAAL** 82

F3 37 PVLGKNTLQSLEKIKTSASVNAKA**AAVVKASALALAEAYLRASALSAAAS** 87

F4 44 GTGASEVASSSGEAIAISLGAGQS**AAESQALAASQSKTAANAAIGASELT** 94

Heptad  *bcdefgabcdefgabcdefgabcdefgabcdefgabcdefgabcdefgab*

F1 63 **DLTAKLSQESASVQSQAAAKGKETEEAAVGQARAGLESVSIAASATSAAK** 112

F2 83 **AAAIAKREEAIKASEIANQLLTNAAKAAEATVSATKRAAQLTAAAKEATR** 134

F3 88 **AKAAAALKNAQQAQLNAQEKSLAALKAQSEEEAASARANAATAATQSALE** 139

F4 95 **NKVAALVAGATGAQARATAASSSALKASLATEEAAEEAEAAVADAKAAAE** 147

Heptad  *cdefgabcdefgabcdefgabcdefgabcdefgabcdefgabcdefgabc*

F1 113 **EASTAAKTAASALSTATVQAKIAERAAKAEAVASEEAKVKAIAAANLAAA** 162

F2 135 **ASAAAAEAATEAQVKANADSIITKRAAIAEAQAAAEAQVKAAIARKSAAN** 182

F3 140 **RAQASSRLATVAQNVASDLQKRTSTKAAAEAAATLRQLQDAERTKWSANA** 187

F4 148 **KAESLAKNLASASARAALSSERANELAQAESAAAAEAQAKTAAAAKAAEI** 194

Heptad  *defgabcdefgabcdefgabcdefgabcdefgabcdefgabcdefgabcd*

F1 163 **ASAAAEAALKAEKVAEEAIARAASAKAAARAAAAALASSKEAATASARNA** 212

F2 183 **FLAKAQIAAAAESEATKLAAEAVVALTNAEVAVNQARNAQANASTQASMA** 234

F3 188 **ALEVSAAAAAAETKTTASSEAANAAAKKAAAIASDADGAERSASTEAQSA** 239

F4 195 **ALKVAEIAVKAEADAAAAAVAAAKARAVADAAAARAAAVNAIAKAEEEAS** 249

Heptad  *efgabcdefgabcdefgabcdefg*

F1 213 **AESEARNEVAVLIAEI**DKKSREID 236

F2 235 **VRVDSQAANAEAAAVA**QAETLLVT 294

F3 240 **AKIESVAAAEGSANSA**SEDSRAAQ 303

F4 248 **AQAENAAGVLQAAASA**AAESRAAA 308

**Supplementary Fig.6:** Heptad assignments from tetrameric coiled coil structure overlayed onto honeybee silk protein sequence alignments from Sutherland et al., 2006; Campbell et al., 2014. The N- and C-terminal sequences are not shown as they cannot be convincingly aligned. Yellow highlight shows coiled coil domain identified in this study. Bold indicates MARCOIL predicted coiled coil domain from bioinformatic assessment of protein sequences (Sutherland et al., 2006; Campbell et al., 2014).


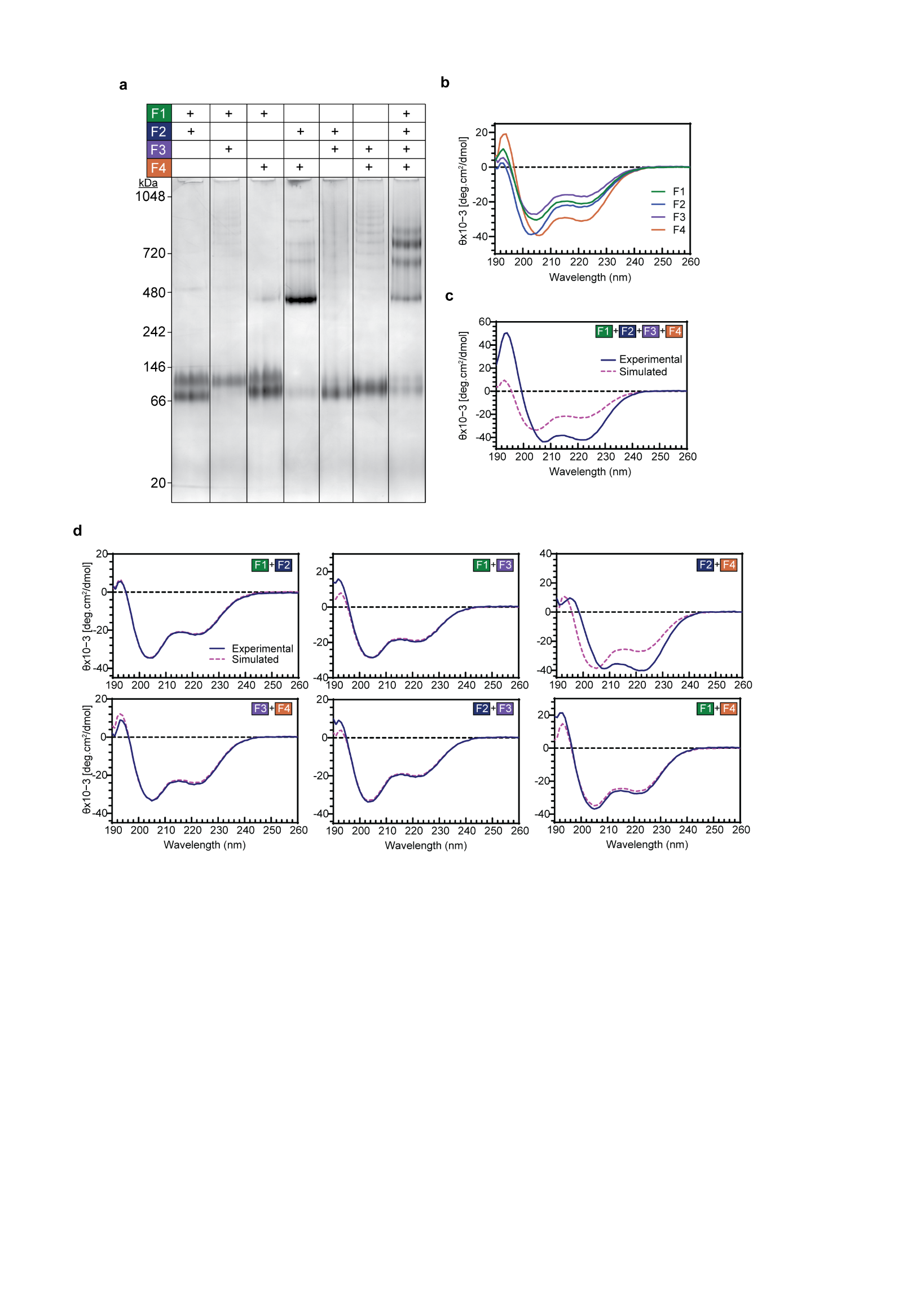


**Supplementary Fig.7:** **Replicate analysis of initial coiled coil silk formation pathway using BN-PAGE and CD.** (**a)** BN-PAGE analysis of silk proteins in mixtures. CD spectra of **(b)** silk proteins alone, **(c)** in a mixture containing all four proteins or (**d)** binary mixtures. Data includes experimental spectra (*blue*) compared to simulated spectra derived from spectra for individual proteins combined (*pink dashed*).


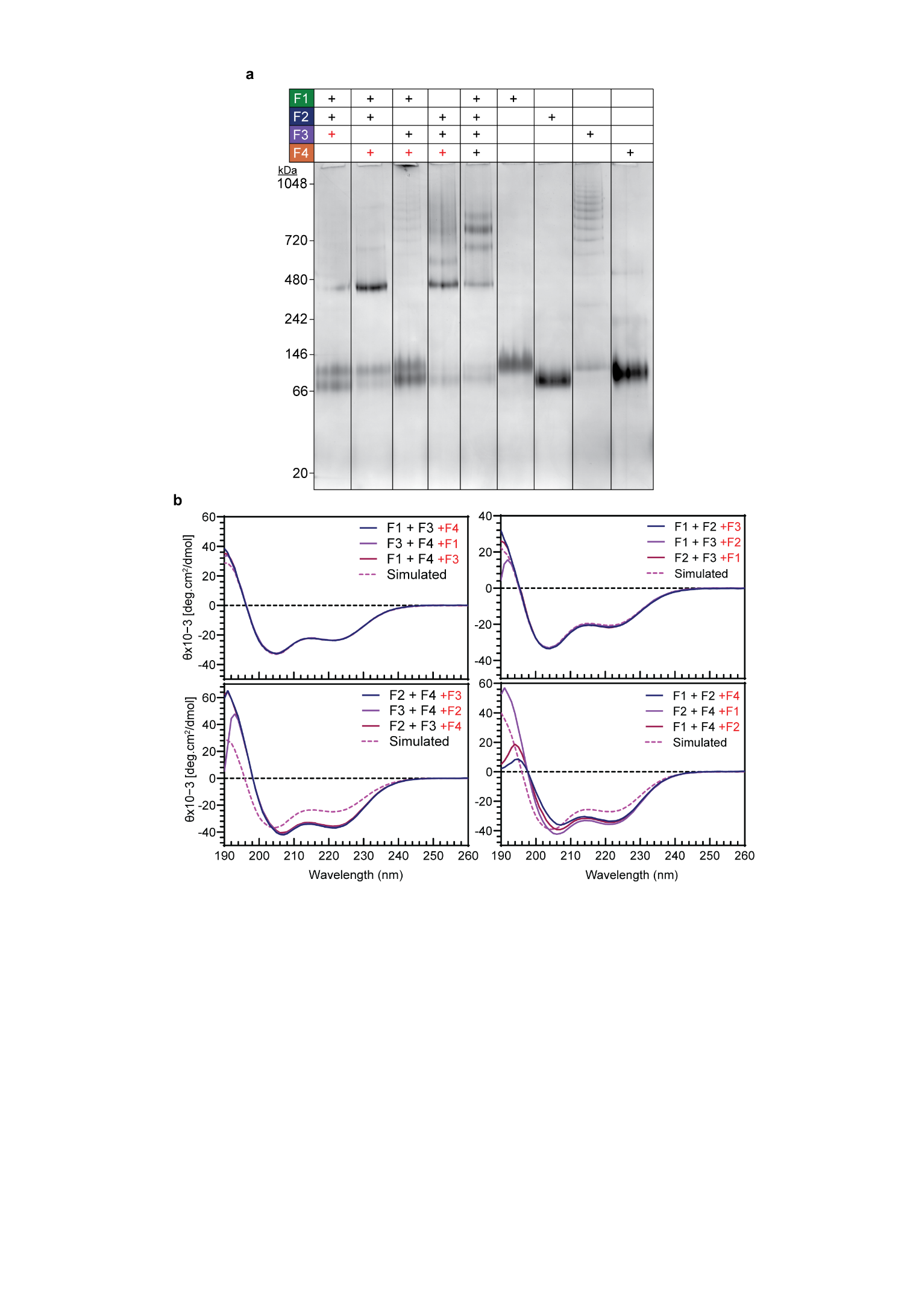
**Supplementary Fig.8:** **Replicate analysis of intermediate structures during the coiled coil formation pathway using BN-PAGE and CD. (a)** BN-PAGE of silk protein solutions. Silk protein indicated with black (+) were initially incubated together before the addition of the final protein indicated with a red (+). **(b)** CD spectra from tri-silk protein solutions. Data includes experimental spectra compared to simulated spectra derived from spectra for individual proteins combined (pink dashed).


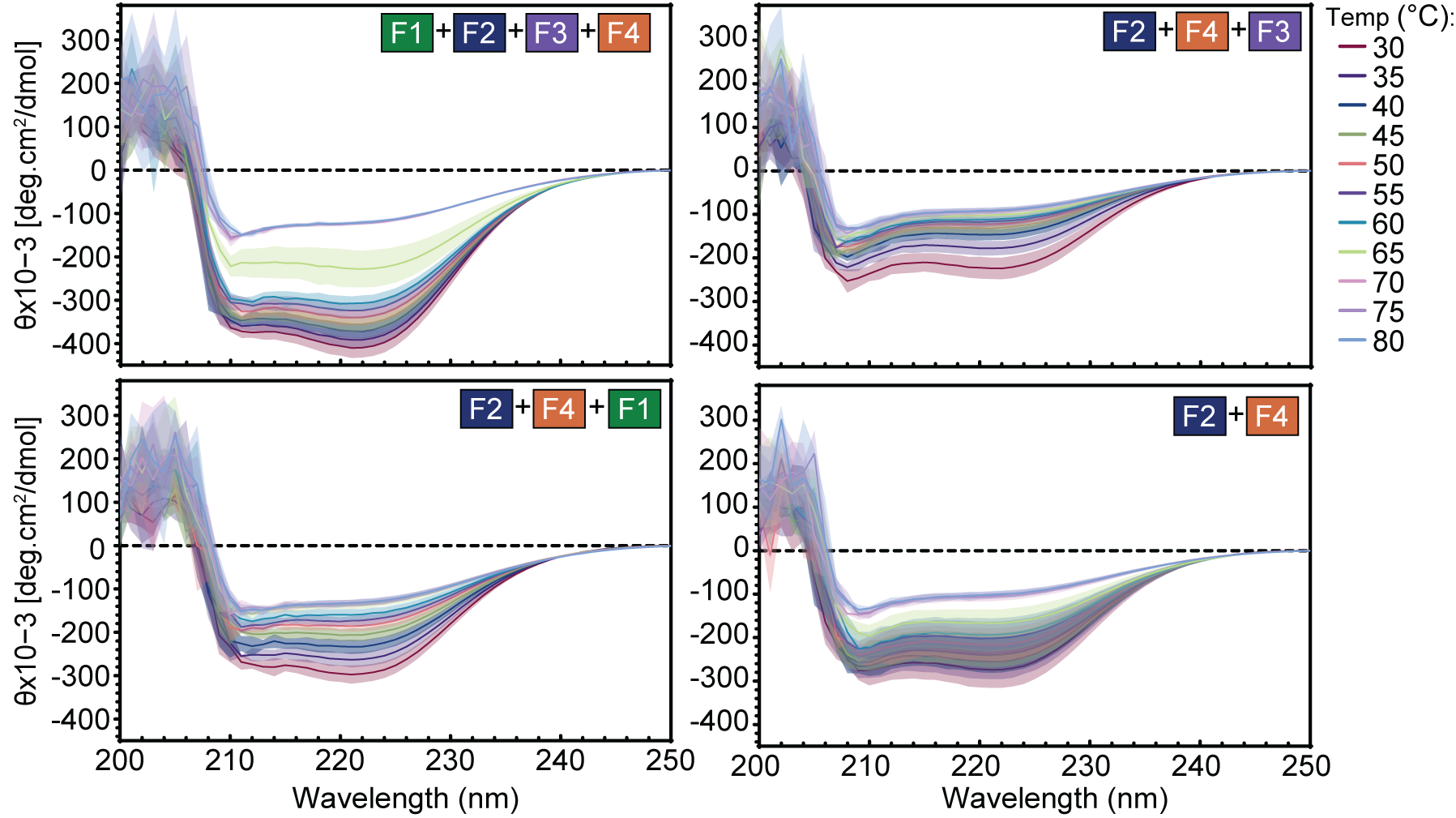


**Supplementary Fig.9:** **Thermal melting CD of silk protein mixtures.** Spectra of each protein mixture at increasing temperature. Data reported as mean +/- standard deviation (shaded area) for each temperature point (n=3).

**References:**

Campbell, P.M., Trueman, H.E., Zhang, Q., Kojima, K., Kameda, T., Sutherland, T.D., 2014. Cross-linking in the silks of bees, ants and hornets. Insect Biochemistry and Molecular Biology 48, 40–50. https://doi.org/10.1016/j.ibmb.2014.02.009

Sutherland, T.D., Campbell, P.M., Weisman, S., Trueman, H.E., Sriskantha, A., Wanjura, W.J., Haritos, V.S., 2006. A highly divergent gene cluster in honey bees encodes a novel silk family. Genome Research 16, 1414–1421. https://doi.org/10.1101/gr.5052606

Williams, C.J., Headd, J.J., Moriarty, N.W., Prisant, M.G., Videau, L.L., Deis, L.N., Verma, V., Keedy, D.A., Hintze, B.J., Chen, V.B., Jain, S., Lewis, S.M., Arendall III, W.B., Snoeyink, J., Adams, P.D., Lovell, S.C., Richardson, J.S., Richardson, D.C., 2018. MolProbity: More and better reference data for improved all-atom structure validation. Protein Science 27, 293–315. https://doi.org/10.1002/pro.3330
